# Protocol for non-invasive assessment of skeletal muscle structure and function in adolescents with single ventricle heart disease: a cross-sectional, case-control study

**DOI:** 10.3389/fcvm.2026.1781505

**Published:** 2026-04-07

**Authors:** Tyler A. Fick, Trent J. Herda, Emily Cramer, David A. White, Daniel Forsha

**Affiliations:** 1 Ward Family Heart Center, Children's Mercy Kansas City, Kansas City, MO, United States; 2 School of Medicine, University of Missouri – Kansas City, Kansas City, MO, United States; 3 Department of Health, Sport, and Exercise Sciences, University of Kansas, Lawrence, KS, United States; 4 Children's Mercy Kansas City, Kansas City, MO, United States

**Keywords:** congenital heart disease, exercise, Fontan, single ventricle (SV), skeletal muscle pump

## Abstract

**Background:**

Congenital heart disease (CHD) is the most common congenital malformation, and the most severe type, single ventricle (SV) heart disease, requires the Fontan surgical palliation. Fontan palliation minimizes hypoxemia and volume overload by separating systemic and pulmonary circulations, resulting in the absence of a sub-pulmonary pump. Skeletal muscle health may have a greater relative contribution to overall functional capacity of those with SV physiology, via the skeletal muscle (SkM) pump mechanism and oxidative capacity. The purpose of this study is to comprehensively evaluate multiple domains of SkM in this population compared to those with normal cardiac anatomy.

**Methods:**

Forty SV patients aged 12–21 years old and 40 matched controls will be recruited. During their single study visit, testing will include an echocardiogram with strain analysis and cardiopulmonary exercise testing by cycle ergometry. SkM domains will be evaluated via ultrasound, advanced near-infrared spectroscopy, and biodex testing. Frailty assessment will also be performed. At the conclusion of the study visit, participants will be equipped with a 7-day device-based physical activity accelerometer.

**Discussion:**

This study is the first to completely evaluate the SkM domains in adolescents with Fontan. The study aims to determine the differences in SkM in SV patients vs. matched controls. Additionally, the relationship between SkM and measures of cardiac function, fitness, and frailty will be evaluated. This study will lay the groundwork for integration of these SkM domains into future SV exercise and outcomes trials.

**Trial registration:**

This study is not a clinical trial study design and was not registered.

## Highlights

- This is an integrated, multi-domain approach to evaluating skeletal muscle in patients with single ventricle palliation. This multi-domain skeletal muscle testing framework is new in this patient population.
- This study will evaluate the hypothesis that adolescents with single ventricle physiology have diminished skeletal muscle health across multiple domains compared to matched adolescents without heart disease.
- This study protocol will evaluate for independent relationships between skeletal muscle domains and both cardiorespiratory fitness and cardiac function.

## Background

Congenital heart disease (CHD) is the most common congenital malformation, affecting ∼1% of all births in the United States (1). Some of the most severe forms of CHD, such as hypoplastic right or left heart syndromes, require three surgical procedures in early life, concluding with the Fontan palliation. The Fontan palliation creates a physiology that relies on functional single ventricle (SV). However, SV cardiac physiology is also associated with several comorbidities, including elevated rates of hospitalization, transplant, and mortality relative to other CHDs (2–5).

The Fontan palliation separates the systemic and pulmonary circulations by surgically connecting the superior and inferior vena cava directly to the pulmonary arteries, resulting in absence of a pump to drive pulmonary flow. Instead, systemic venous return of deoxygenated blood passively flows to the lungs prior to returning to the heart (6). This passive pulmonary return is a functional limitation of SV cardiac physiology, where downstream venous filling of the systemic SV and stroke volume are suppressed, leading to impairments in functional and exercise capacity and elevated physical fatigue (7, 8).

The skeletal muscle (SkM) system is broadly understood to be a key mediator of passive pulmonary return augmenting exercise capacity in SV cardiac physiology through three primary physiologic domains: 1) the SkM pump (SkMP) mechanism, 2) the SkM aerobic or oxidative mechanisms, and 3) the SkM anaerobic and neuromotor mechanisms. First, the SkMP relies on muscle tone, volume, and tension of contraction to compress vascular beds in the lower extremities and promote flow of blood into the central vasculature (9). The SkMP is an important facilitator of systemic venous return and pulmonary blood flow in the absence of a sub-pulmonary ventricle, thereby driving downstream filling of the SV and ultimately cardiac output (10, 11). Second, efficiency of oxygen utilization or aerobic capacity in SkM has significant effects on energy, fatigue, and functional capacity at rest, during activities of daily living, and with exercise. Fick's principle describes that aerobic energy production (oxygen consumption) is determined by arterial-venous oxygen difference and cardiac output (12). In the SV population where cardiac output is limited and hypoxemia is common, the efficiency of the SkM's capacity to produce energy in an aerobic state could play a more prominent role in the Fick principle than cardiac output. Lastly, SkM weakness is prevalent in the SV population and has been correlated with low exercise tolerance, quality of life, and impacts generalized fatigue with activities of daily living (13, 14).

The SkM has a greater relative contribution to the overall functional capacity of those with SV physiology than those with normal cardiac physiology. However, research comprehensively studying the SkM in those with SV physiology remains incomplete. The purpose of this study is to systematically evaluate and describe the SkM system through four SkM domains using non-invasive methods in adolescents with SV physiology: 1) cross-sectional area, quality, volume; 2) oxidative capacity; 3) strength; 4) motor unit (neural) activation.

### Objectives

Primary Objective: To assess the differences in the SkM between adolescents with SV physiology and healthy controls matched by age, sex, body mass index, and physical activity (PA). We hypothesize that adolescents with SV physiology have diminished SkM health across all four domains compared to matched adolescents without heart disease.
Secondary Objective: To evaluate the independent relationships between SkM domains and both cardiorespiratory fitness and cardiac function. We hypothesize that modeling these relationships while controlling for appropriate confounders will identify SkM domains that have independent effects on cardiorespiratory fitness and cardiac function.
Exploratory Objective: To explore the relationship between SkM domains and physical frailty. We hypothesize that deficiencies in specific SkM domains will impact the development of physical frailty.

## Methods/design

### Design

This investigation, referred to as the “Fontan Muscle Study,” will follow a cross-sectional cohort design. Adolescents with SV cardiac physiology who have had Fontan palliation are considered the exposed cohort (henceforth referred to as “SV group”), and healthy cardio-typical adolescents (without heart disease) are considered the unexposed cohort (henceforth referred to as the “control group”). The SV group will be recruited first, and after 65% of the SV sample has been enrolled, recruitment of the control group will commence. Data collection will be performed at a single time-point, except for the device-based measures of PA, which will be assessed across the 7 days following participants’ single-day study visit.

### Study setting and recruitment

All data collection will be performed at the Adele Hall Campus of Children's Mercy Kansas City Hospital (CMHKC) and the collaborative Center for Children's Healthy Lifestyles & Nutrition (CCHLN), both part of CMHKC and located in Kansas City, Missouri, USA. All study visits will occur in person and will be supervised by a trained member of the Fontan Muscle Study research team.

Participants will include local and regional population of adolescent patients with SV cardiac physiology who are referred to or followed by the Heart Center at CMHKC. Potential participants will be identified through automated reports derived from querying upcoming patient visits, medical record review, or referral by their primary cardiologist. Additionally, flyers, emails, mailings to physical addresses will provide information for adolescents/families to self-refer to the study who may not be regularly followed at CMHKC. Lastly, the study materials and flyers will be provided to local and regional private pediatric cardiology practices, local non-profit organizations that serve families of children with CHD, the CMHKC Heart Center Parent/Family Advisory Council, and parent/family groups. Control group participants will be recruited from the greater Kansas City community through flyers and partnerships with primary care pediatricians. This study was approved by the IRBs at CMHKC and the University of Kansas.

### Screening and matching

Potential participants in the SV group will be added to a screening database. Screening procedures will include reviewing the medical record with the primary cardiologist to identify abnormalities from prior clinical assessments that would deem participation unsafe, as well as any disabilities that would inhibit their ability to complete study procedures. Potential participants will be screened out if their prior CPET and/or echo testing has the below findings. CPET abnormalities include significant chronotropic deficiencies [as defined as peak heart rate achieved of less than 80% of maximum predicted peak heart rate (15, 16)], hypoxia during exercise (change greater than 10% from baseline), blunted or decreasing blood pressure during exercise, ST-segment depression ≥3 mm, severe hypertension (defined as systolic blood pressure >250 mmHg or diastolic blood pressure >125 mmHg) (17). Recent echocardiographic abnormalities such as moderate-severe or greater ventricular dysfunction, severe atrioventricular valve regurgitation, or significant aortic arch obstruction will also result in those potential participants being screened out. Those who have a history of taking insulin, growth hormone or have other disease processes with primary muscle effects (e.g., muscular dystrophy, genetic short stature) will be excluded. No additional clinical variables will be included; however, each patient will be reviewed with the primary cardiologist to ensure clearance for participation.

Control group participants will be screened for eligibility. To facilitate matching, control group participants who are interested in participating in the study will be required to complete an online assessment via REDCap where they will be asked to self-report age, height, weight, sex, history of cardiac diagnoses, and PA participation (assessed by the Physical Activity Questionnaire for Adolescents (PAQ-A, +/− 0.5). Control group participants will be prospectively matched to SV group participants.

### Study participants

#### Inclusion and exclusion criteria

Inclusion and exclusion criteria for the SV group and control group are displayed in Table 1 and as discussed above. Briefly, SV adolescents who have undergone a Fontan palliation with any type of underlying cardiac morphology are eligible for the SV group. Patients who have had Fontan palliation and subsequently been converted to biventricular physiology will not be eligible.

**Table 1:** Inclusion and exclusion study criteria.

Inclusion Criteria	Exclusion Criteria
• Adolescents, age 12 to 21 years old • Male or female • Capable of providing informed consent or assent, requiring a parent or LAR for potential participants <18 years old. • **SV Group:** o SV physiology following Fontan palliation o Clearance for study participation from the primary cardiologist • **Control Group:** o Normal cardiac anatomy^c^	• Height >132 cm • Pregnancy • Violating any safety criteria from CPET^a^ or inability to achieve maximal effort^b^ on CPET • Evidence of insulin resistance or progressive muscle disorders • History of hormone replacement therapy • Severe symptoms related to circulatory failure • Moderate-severe or worse ventricular dysfunction, severe valve dysfunction, significant arch obstruction

LAR, legally authorized representative; SV, single ventricle; CPET, cardiopulmonary exercise test.

^a^ CPET safety criteria are described in the Measures section under CPET.

^b^ Maximal effort defined as respiratory exchange ratio (RER) ≥1.1.

^c^ Criteria used to define normal cardiac anatomy are listed in the inclusion and exclusion criteria section.

The control group participants are required to have normal cardiac anatomy, defined as four-chambered heart without evidence of chamber enlargement or hypertrophy, with proper atrioventricular and ventriculoarterial connections, intact atrial and ventricular septa, and normal cardiac valves. Participants who meet eligibility criteria following screening procedures and enroll in the study may be excluded if any unanticipated abnormalities that either violate inclusion criteria or satisfy exclusion criteria are identified during outcomes testing. Participants unable to complete the full cohort of testing will also be excluded.

Parents or legally authorized representatives (LARs) are also considered research participants as they are asked to participate in collection of some demographic and patient-reported data. Participation of a parent or LAR is required for adolescent participants <18 years old. Parents or LARs are not required to participate for adolescent participants ≥18 years old.

#### Sample size

The study sample will consist of 40 adolescents in the SV group and 40 adolescents in the control group, for a total of 80 participants. The distribution of the SV group will not be stratified by underlying cardiac morphology however SV morphology will be recorded. The planned enrollment of 80 participants will adequately power this study for aims 1 and 2. Sample size adequacy for aim 2 was assessed using a simulation study. Assuming a normally distributed focal explanatory variable, 7 multivariate normal covariates with pairwise correlation r = 0.25, R-squared = 0.20 for regression of the focal explanatory variable on covariates, and standardized regression coefficient = 0.40 for the focal explanatory variable, 10,000 data sets were simulated of size *n* = 80. For each data set, a linear model was fit to estimate the focal variable's standardized regression coefficient and compute its 95% confidence interval (CI). The mean margin of error (half the width of the 95% CI) for these estimates equaled 0.19, and 98% of CIs were entirely above 0, which is equivalent to 98% power in a hypothesis testing framework. These simulations for aim 2 will result in a more than adequate sample size for the models in aim 1, where sample size per model explanatory variable will be much greater.

### Measures and data collection

Primary and secondary outcome measures are summarized in Table 2. Each outcome measured is described in greater detail below.

**Table 2:** Primary and secondary outcome measures.

Outcomes	Measures
**Primary Outcomes**	
SkM cross-sectional area/Volume	SkM Ultrasound: total cross-sectional area (volume); mean echo intensity (quality)
SkM quality	
SkM oxidative capacity	aNIRS: exponential recovery rate time-constant
SkM strength	Isokinetic testing and nerve stimulation: voluntary leg extensor strength and
Non-voluntary (neural activated) leg extensor strength; peak twitch torque per cross-sectional area	
SkM motor unit activation	
**Secondary Outcomes**	
Cardiac Function	Echocardiogram: GLS
Cardiorespiratory Fitness	CPET: VO_2peak_
Body Composition	DXA: Lower body lean body mass volume
PA	Accelerometry: minutes of moderate-to-vigorous intensity PA
**Exploratory** **Outcomes**	
Physical Function	Frailty: Composite frailty score
Patient Reported Outcomes	PedsQL: QoL overall and sub-domain scores
PA self-efficacy	Domain-Specific Physical Activity Efficacy Questionnaire: raw scale score

SkM, skeletal muscle; aNIRS, advanced near-infrared spectroscopy; GLS, global longitudinal strain; CPET, cardiopulmonary exercise test; DXA, dual-x-ray absorptiometry; QoL, quality of life; PA, physical activity.

#### General procedure

**Figure 1:**
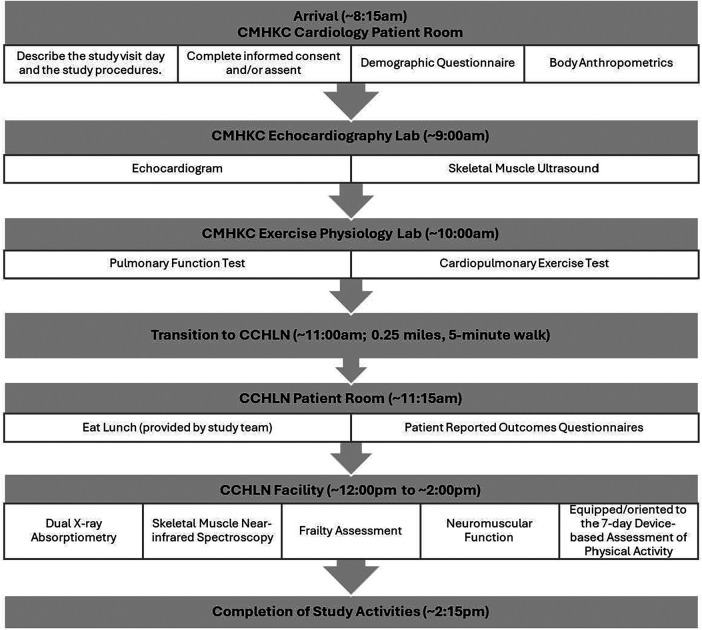
Standardized order of outcomes testing. CMHKC, Children's Mercy Kansas City Hospital; CCHLN, Center for Children's Healthy Lifestyles & Nutrition.

Participants will be scheduled for a 5–6-hour single-day visit. Written informed consent/assent will be obtained prior to data collection. A study team member familiar with the study protocol and procedures will accompany the participant(s) throughout the visit. The standardized order of outcomes testing during the study visit is displayed in Figure 1. A 6-hour day of testing could potentially limit recruitment, but we expect to achieve a 40% enrollment rate based on past experience in our Fontan cohort and 15% enrollment rate for the matched control cohort. The plan is to screen over 100 potential Fontan participants and over 300 potential matched control participants in total to achieve our target enrollment of 40 Fontan and 40 controls. The study protocol indicated in Figure 1 has been designed to chronologically allow for minimum fatigue-related measurement variability, allowing several hours and a provided lunch between CPET and frailty and neuromuscular function testing. Participants who do not complete a maximal CPET, muscle testing and echo will be excluded from the study.

#### Demographics, body anthropometrics, and pubertal status

Study personnel will collect demographic information during their interview with the participant and/or parent/LAR. Descriptive and demographic questions are described in Table 3. Height will be measured to the nearest 0.5 cm and weight will be measured to the nearest 0.1 kg. Height and weight will be used to calculate BMI using a standard equation, and BMI percentile and z-score will be derived using age- and sex-specific normative data (18). Waist circumference will be measured immediately above the iliac crest to the closest 0.5 cm and used to calculate waist-to-height ratio. Age- and sex-specific waist circumference and waist-to-height ratio percentiles and z-scores will be calculated using normative data (19). Percentiles and z-scores for participants older than the available normative data will not be calculated.

**Table 3:** Descriptive and demographic variables.

Variable	Response options for categorical variables	
Sex at birth	• Male	• Female
Race	• White	• LatinX
	• Black or African American	• Native Hawaiian/Pacific Islander
	• Asian	• Multi-racial (select multiple options)
	• American Indian or Alaska Native	
Ethnicity	• Hispanic	• Unknown
	• Non-Hispanic	
Insurance status	• Yes	• No
Type of insurance	• Private	• Public & Private
	• Public	• Unknown
Parents’ highest level of education	• K-6th	• Partial college/2-year/trade
	• 7th−9th	• 3- or 4-year college
	• 10th−11th	• Post-graduate
	• High School level graduate	• Declined to answer
Home zip code		

Pubertal maturation will be assessed with the pubertal self-assessment survey, a self-report tool previously used for the assessment of pubertal maturation in CHD populations (20). This self-assessment tool has been used for SV patients in other comparable studies (20, 21). The adolescent participant can choose to complete the survey themselves or with the help of a parent/LAR.

#### Echocardiogram

**Figure 2:**
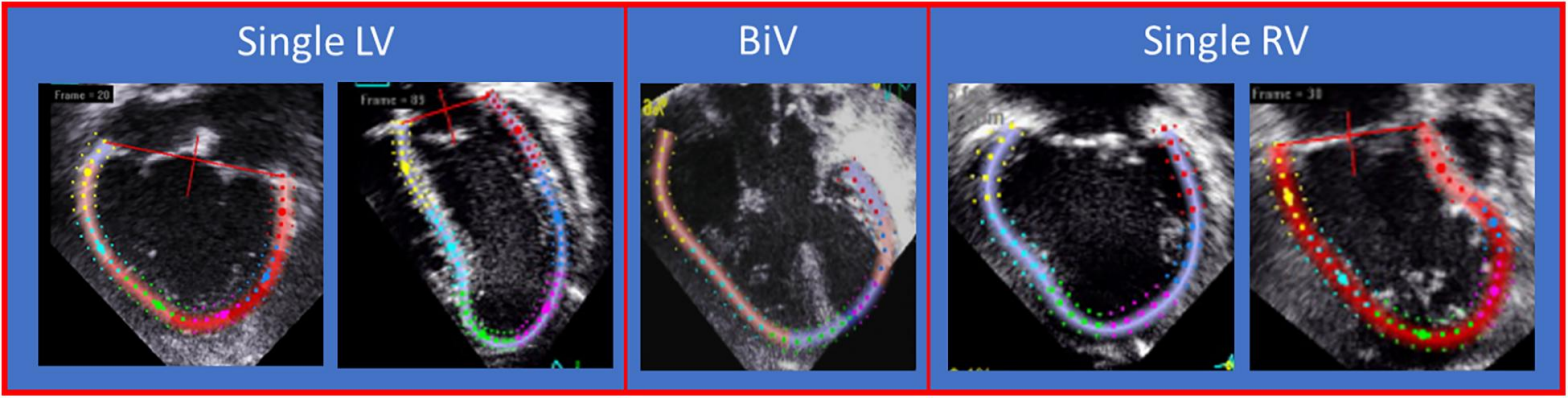
Apical view showing different examples of SV strain tracking. The leftward two panels labeled “single left ventricle” show double inlet left ventricle and tricuspid atresia morphologies. The middle two panels labeled “single right ventricle” show examples of strain tracking for double outlet right ventricle with mitral atresia and hypoplastic left heart syndrome morphologies. The right panel labeled “BiV” shows an example of strain tracking of an unbalanced atrioventricular canal. BiV, Biventricular heart.

Echocardiograms will be performed by trained sonographers as part of the CMHKC Cardiac Imaging Research Core Lab using a GE Vivid E9 (GE Healthcare, Milwaukee, WI, USA). The echocardiograms will include assessment of SV morphology as well as global longitudinal strain (GLS) using GE Viewpoint (6.11.3, GE Viewpoint, Milwaukee, WI, USA), and the echocardiograms will be analyzed at the time of assessment by study investigators with GLS expertise (22, 23). For SV group, apical “four-chamber” images optimized for strain analysis will be obtained with left- and right-sided walls within the sector arc. Single ventricular GLS will be measured following American Society of Echocardiography guidelines modified to track left- and right-sided walls that produce ventricular ejection based on ventricular morphology of the single ventricle anatomy (Figure 2) (24). Targeted echocardiogram outcomes for the SV group include SV GLS, obstruction in Glenn, Fontan, branch pulmonary artery flow, residual coarctation, or valve dysfunction.

For control participants, an echocardiogram will be performed to assess ventricular function (primary outcome left ventricular GLS), valve function, septa, branch pulmonary arteries, and arch. Significant abnormalities in any of these domains will lead to the participant being excluded from the study.

#### SkM ultrasound

Ultrasounds of the SkM will be performed at the time of echocardiogram by sonographers who have been trained on SkM assessment. Ultrasound has been found to be a reliable, non-invasive, validated approach for assessment of SkM cross-sectional area and SkM quality that accounts for the non-contractile adipose and connective tissue present within the area of the muscle (25). Imaging of the quadriceps muscle will be performed, as described in the Supplementary Material. Primary outcomes of SkM ultrasound are total cross-sectional area (cm^2^) and mean echo intensity (AU).

#### CPET

Prior to the CPET, participants will perform a minimum of three rounds of pulmonary function testing on Jaeger Medical (Höchberg, Germany) Vyntus Spiro PC spirometer using Sentry Suite version 3.10. Pulmonary function testing will permit assessment of breathing reserve (26). Participants will be equipped with a 12-lead ECG using GE CASE ECG System (GE Healthcare, Wauwatosa, WI, USA), and oxygen consumption will be measured using Parvo Medics (Salt Lake City, UT, USA) TrueOne 2400 breath-by-breath oxygen analyzer with Hans Rudolph (Kansas City, KS, USA) oro-nasal facemask and two-way non-rebreathing valve. Blood pressure and oxygen saturation will be assessed throughout the CPET. Cardiorespiratory fitness (VO2peak) will be assessed using CPET on a Lode Corival CPET electronically braked cycle ergometer (Gronigen, Netherlands) with a commonly used ramp protocol (27). The protocol consists of the following activities completed in order: 1) 3 min of seated, resting assessment of oxygen consumption; 2) a 3-minute warm-up consisting of pedaling at a target of 75–85 rpm with no resistance; 3) pedaling continuing at 75–85 rpm, with resistance added at each 1-minute interval based on participant weight (resistance (watts) = (body weight × 3)/10); and 4) when the participant is nearing volitional fatigue, a “sprint” where they pedal as fast as they can for as long as they can. VO2peak is defined as highest oxygen consumption (L/min) at an RER ≥1.1. The peak exercise test will permit analysis of both peak and sub-peak measures of exercise oxygen kinetics such as anaerobic threshold, oxygen pulse which may be valuable to Fontan patients (26, 28). Peak and sub-peak CPET outcomes will be analyzed as percent of predicted based on age, sex, and race using the normative data for this cycle protocol (29). Participants will be permitted one opportunity to repeat the exercise test (within 5 days) if an RER ≥1.1 is not achieved on the first attempt.

**Figure 3:**
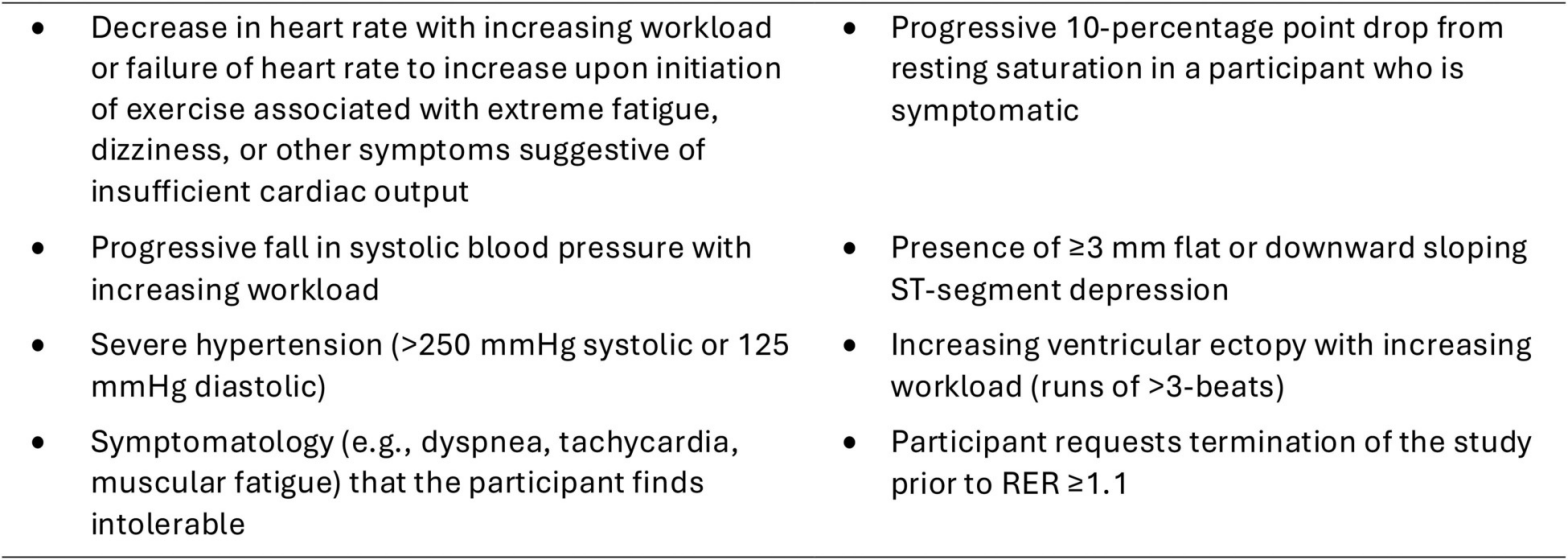
CPET safety criteria. CPET, cardiopulmonary exercise test; RER, respiratory exchange ratio.

The CPET safety criteria are described in Figure 3 (30). The CPET will be immediately terminated if any of the CPET safety criteria are not met. This test will be performed by trained exercise physiologists at CMHKC. The CPETs are administered by exercise physiologists who are trained and have expertise in pediatric and CHD exercise testing.

#### Patient-reported outcomes

Self-report and parent-proxy quality of life (QoL) and self-efficacy of PA will be assessed using validated questionnaires/surveys. Measures of generic/global and disease-specific QoL were self-reported using the Pediatric Quality of Life Inventory (PedsQL™) Generic Core Scale v4.0 for teens and the PedsQL™ Cardiac Module v3.0 for teens (31, 32). The PedsQL™ Generic Core Scale is subdivided into four subscales focused on physical aspects, emotional aspects, school, and social functioning. The PedsQL™ Cardiac Module is subdivided into six subscales focused on heart problems and treatment, treatment specific to heart medications, perceived physical appearance, treatment anxiety, cognitive problems, and communication. Both scales asked participants to respond to statements representing the past month. Descriptive responses aligned with numeric values and were reverse scored and summed to elucidate an overall summary score and subscale scores as described in the scoring manual with a range from 0 (lowest QoL) to 100 (highest QoL) (33). Self-efficacy of PA will be assessed using an abbreviated version of the Domain-Specific Physical Activity Efficacy Questionnaire (34). The participant self-report measure focuses on self-efficacy related to household PA and leisure and recreational PA.

All questionnaires and surveys will be checked by the research coordinator for completeness prior to end of the study visit and participants and/or parents/LARs will be asked to complete any incomplete items.

#### Dual x-ray absorptiometry (DXA)

A full-body DXA scan will be performed using GE Lunar DPX DEXA scanner system, Version 3.6; Lunar Radiation Corp (Madison, WI, USA) for assessment of body composition. Specific DXA outcomes of interest include lower body lean mass as this has been associated with blood flow in patients with Fontan and total cavopulmonary connection (22, 35). Lower body lean mass (kg) will be normalized to a z-score using published normative reference data and adjusted for leg-length z-score (36). All female participants will be required to have a negative pregnancy test prior to completing the DXA scan.

#### SkM oxidative capacity

SkM oxidative capacity, the rate at which the muscle can utilize oxygen to meet increases in energy demand, will be assessed using near-infrared spectroscopy (NIRS) and a standardized protocol (37). Assessments will be performed by trained research associates and a study investigator using ISS Medical (Champaign, IL, USA) Dual-Channel OxiplexTS advanced NIRS (aNIRS) device. The OxiplexTS device uses frequency domain technology to provide an absolute determination of tissue oxy[heme] and deoxy[heme]. The aNIRS sensor is positioned longitudinally on the belly of the right VL muscle with a blood pressure cuff positioned proximally. While supine, participants will kick against a TheraBand attached to the assessment table for 10–15 s to increase oxygen consumption of the VL. Following voluntary contraction, the participants will be asked to relax their leg fully and recovery kinetics of the muscle are measured using a series of 10 transient arterial occlusions (rapid inflation cuff; 5 s on/5 s off) followed by a series of 10 transient arterial occlusions (10 s on/10 s off). Following each occlusion, the rate of change (slope) in the decline in deoxy[heme] is calculated by specialized software providing the time-constant variable representing oxidative capacity and an R-squared representing appropriateness of the fit of the curve (test quality). The procedure will be performed twice and the results will be averaged. If significant variability in the time-constant between the two tests is observed, or if one of the tests produces an R-squared <0.7, a third test will be performed, and the best two measures will be averaged. The primary outcome of this assessment is single value exponential recovery rate time-constant representing an estimate of SkM oxidative capacity.

#### Frailty assessment

Frailty represents comprehensive physical dysfunction across multiple domains. The frailty protocol for youth with CHD has been previously described (38, 39). Briefly, five independent physical domains will be assessed: 1) Slowness (6-minute walk test) (40); 2) Weakness (handgrip dynamometry) (41, 42); 3) Exhaustion (PedsQL™ Multidimensional Fatigue Scale) (43); 4) Body composition (DXA); and 5) Diminished PA (PAQ-A) (44). Self-report PA will be estimated using the PAQ-A (44–46). The 8-item PAQ-A is designed to assess participation in both recreational and school-based PA over the past 7 days. The PAQ-A summary score is an average of each survey item ranging from 1 (lowest activity) to 5 (highest activity).

Age- and sex-specific Z-scores will be assigned based on outcomes of the 6-minute walk test (40), handgrip dynamometry (47), and lean-mass component of the DXA (48). These Z-scores combined with scores from the Multidimensional Fatigue Scale and PAQ-A will be individually scored and aggregated into a composite frailty score as described previously (38). Trained research personnel will administer the 6-minute walk test and handgrip strength test, while other frailty domains will be assessed using previously described methods. The primary outcome of the frailty assessment is the composite frailty score which ranges from 0 (least frail) to 30 (most frail). The frailty composite score will be compared between Fontan and matched control cohorts, and modeling will evaluate for a relationship between frailty and the SkM testing variables.

#### Neuromuscular function

Leg extensor neural activation and strength will be assessed with isokinetic testing and nerve stimulation of the femoral nerve at rest, serving as a method to assess neuromuscular function (49). Detailed methodology is highlighted in detail in Supplementary Material. Participants will complete a familiarization session prior to experimental data collection as previously described (49). For non-voluntary leg extensor strength, transcutaneous stimuli are applied to the femoral nerve using a constant current stimulator (Digitimer DS7AH, Hertfordshire, United Kingdom) (50). The primary outcome is neural activation of the SkM, and voluntary and non-voluntary leg extensor strength measured in peak twitch torque per cross-sectional area (Nm/cm^2^).

#### Device-based assessment of PA

Free-living PA will be assessed using belt-worn ActiGraph wGT3X-BT accelerometer (ActiGraph LLC, Pensacola, FL) on the right side of the hip over a consecutive 7-day period. At the conclusion of the visit, participants will receive their monitor, with detailed participant instruction guide, a monitor diary sheet, and a pre-paid self-addressed padded envelope for return of the device. Participants will be equipped with the monitor in the correct position prior to concluding the visit and instructions will be given by a trained member of the team.

Free-living PA data of returned accelerometers will be analyzed by the CMHKC Field-Based Physical Activity Measurement Core. Activity intensity, including sedentary, light, moderate, and vigorous intensity PA will be scored using accelerometer PA intensity cut-points (51). Bouts of moderate-to-vigorous intensity PA will be analyzed using methods previously described (52, 53).

#### Feasibility and safety

The feasibility of the study will be based on safety and completion of testing. The research team will track the number of participants who are not able to complete all intended tests. The research team will assess for any adverse events experienced by participants. If any abnormalities are observed, participants will be referred to their cardiologist or a specialist for follow-up. A pediatric cardiologist from the research team will be available on campus to provide medical oversight for testing appointments for participants in the SV group. All participants that proceed to the CCHLN will have successfully completed the CPET without early termination or any of the findings described in Figure 3. Participants can request to terminate any test at any time. Any of the following will result in immediate termination of the testing: chest pain, dizziness/lightheadedness, syncope, nausea or vomiting, headache, persistent shortness of breath, and feelings of irregular heartbeats.

#### Data analysis and management

Between-group differences in the SV group and controls for the primary SkM measures will be evaluated. Assessment of differences in primary SkM measures will include 1) time-constant (oxidative capacity, aNIRS), 2) cross-sectional area/echo intensity (size/quality ratio, ultrasound), 3) firing rates (neural activation, EMG), 4) y-intercept and slope (voluntary strength, leg extension), 5) peak twitch torque (NerveStim) by modeling each variable as a function of group (SV vs. control). Propensity score weighting will adjust for any between-group imbalances on BMI z-score, Tanner stage, and PA. Separate assessment of the relationships between primary SkM measures and dependent variables of cardiorespiratory fitness (VO2peak) and cardiac function (GLS) adjusting for growth, pubertal status, and cardiac disease will be performed. To limit the number of variables per model and avoid multicollinearity, models will each include one of the primary SkM variables, along with the following covariates: age, sex, Tanner stage, PA, BMI z-score, presence of SV (case vs. control), and SV morphology. Frailty score will be modeled as a function of primary SkM variables, adjusting for the same covariates, including SV morphology and SV GLS, as well as for cardiorespiratory fitness. Due to the small amount of data on SV frailty score, this is an exploratory aim and, as such, the impact of the longer list of covariates on statistical precision will be tolerated and results will be interpreted with caution. To address potentially inflated error rates across the multiple tests, all results, regardless of significance level, will be reported with traditional (unadjusted) *p*-values, 95% confidence intervals, effect size estimates, and Bonferroni-adjusted *p*-values to provide clear interpretabilities for all findings and balance Type 1 and 2 errors.

## Discussion

Patients with SV physiology are at higher risk of compromised SkM health, potentially leading to adverse outcomes. Critical knowledge gaps have been identified including an absence of studies that comprehensively evaluate the pediatric SkM and the impact of each SkM domain on critical measures of overall SV health. Specifically, mitochondrial oxidative capacity, motor unit neural activation, and muscle quality in SV patients have been poorly studied. This proposed study is the first to completely evaluate all aspects of SkM in adolescents with SV physiology and provide an integrated, multi-domain approach. Using this methodology, the study aims will determine the differences in the SkM between SV adolescents and healthy matched controls, as well as differences in SkM function in participants with higher and lower VO2peak. Although the design prohibits identification of causality, the findings will provide valuable insights into mechanisms for future study and intervention.

There are limitations to this study. As a single center study, the sample population may lack external validity to a broader population. Adolescents with more severe disease are unable to complete this testing, and this may bias the results to a healthier SV cohort. Additionally, despite Tanner-stage adjustment, there may be pubertal and post-pubertal confounders we are not able to completely adjust for. Finally, different SV morphologies are included in this study, and this could lead to dilution of more SV domain-specific effects.

This study will evaluate each SkM domain in SV and control patients and will likely generate additional hypotheses. This study may also provide preliminary evidence that supports future specific exercise intervention trials in the SV population (e.g., muscular strength, hypertrophy, activation, and aerobic training). The current study is hypothesis generating for future clinical applications of this data including a potential SkM testing bundle. Relating these findings to health outcomes and determining the validity of such a testing bundle will require further longitudinal and interventional studies to translate these findings into clinical practice.

## Data Availability

The original contributions presented in the study are included in the article/Supplementary Material, further inquiries can be directed to the corresponding author.
