# Supplementary material for: Protocol for non-invasive assessment of skeletal muscle structure and function in adolescents with single ventricle heart disease: a cross-sectional, case-control study
Source: Front Cardiovasc Med. 2026 Apr 7;13:1781505. doi: 10.3389/fcvm.2026.1781505 (PMC13096089; doi:10.3389/fcvm.2026.1781505)
Supplement: Supplementary file 1 [file Datasheet1.docx]

Supplemental File:

*Skeletal Muscle Ultrasound*

Imaging of the vastus lateralis (VL), rectus femoris (RF), and vastus medialis (VM) muscles of the right quadricep will be performed on Logiq Q Ultrasound (GE Healthcare, Wauwatosa, WI, USA) and analyzed using ImageJ software (version 1.46r; National Institutes of Health, Bethesda, MD, USA) to assess for muscle area (cm^2^) and muscle quality via mean echo intensity expressed in arbitrary units (AUs). Ultrasound brightness mode (B-mode), the musculoskeletal preset, and a GE 12L-RS Linear Ultrasound Transducer (5-13 MHz) will be used in accordance with previous work in youth [1,2]. SkM ultrasound images will be read by one of the study investigators and interpreted intermittently throughout the study period. See supplemental figure 4 for sample evaluation of ultrasound performed assessing the rectus femoris, vastus lateralis and vastus medialis (supplemental figure 4).

*Neuromuscular function methodology:*

Maximal strength of the right leg is measured by completing six maximal isokinetic leg extension contractions at three different velocities (1.05 rad·s-1, 2.09 rad·s-1, and 3.14 rad·s-1). Isometric testing (limb remains stationary) is avoided due to a theoretic risk of adverse events with isometric movements in the SV population. Neural activation during the isokinetic testing is measured with Galileo and Avanti surface electromyography electrodes placed on the VL, RF, and VM at the same locations of the SkM ultrasound images (Delsys Inc. Boston, MA, USA). Peak torque from the isokinetic muscle actions is determined from the highest 0.25-second epoch during the isokinetic load range as indicated via the velocity signal. A single stimulus is used to determine the optimal stimulation electrode location (20 mA) and the maximal muscle action potential (M-wave) with incremental increases (2-100 mA). Once a plateau in the peak-to-peak M-wave and torque is determined, 20% will be added to the amperage that yields the highest peak-to-peak M-wave and torque to ensure a supramaximal stimulus. For non-voluntary leg extensor strength, A single stimulus is used to determine the optimal stimulation electrode location (20 mA) and the maximal muscle action potential (M-wave) with incremental increases (2-100 mA). Once a plateau in the peak-to-peak M-wave and torque is determined, 20% will be added to the amperage that yields the highest peak-to-peak M-wave and torque to ensure a supramaximal stimulus. This protocol has been described in previous work [2].

REFERENCES:

1. Herda, T.J., et al., *Examination of muscle morphology and neuromuscular function in normal weight and overfat children aged 7-10 years.* Scand J Med Sci Sports, 2018. **28**(11): p. 2310-2321.

2. Herda, T.J., et al., *Skeletal Muscle Composition and Glucose Levels in Children Who Are Overweight and Obese.* Pediatr Exerc Sci, 2020. **32**(3): p. 157-164.
