# Supplementary material for: Protocol for non-invasive assessment of skeletal muscle structure and function in adolescents with single ventricle heart disease: a cross-sectional, case-control study
Source: Front Cardiovasc Med. 2026 Apr 7;13:1781505. doi: 10.3389/fcvm.2026.1781505 (PMC13096089; doi:10.3389/fcvm.2026.1781505)
Supplement: Supplementary file 2 [file Image1.pdf]

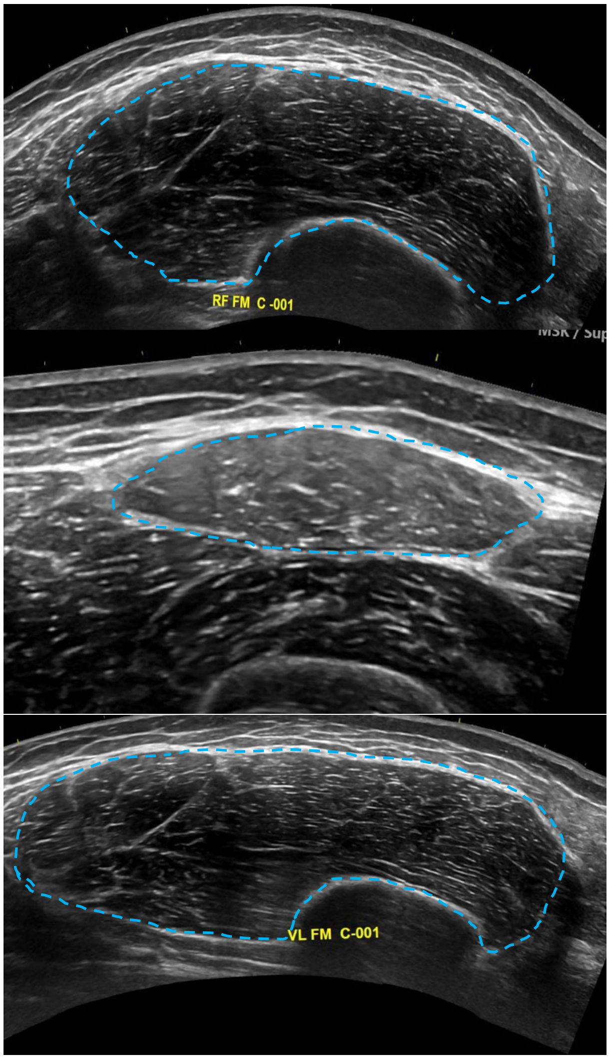

Figure 4: Skeletal muscle ultrasound performed assessing the rectus femoris (top panel), vastus lateralis (middle panel) and vastus medialis (lower panel). The blue dashed line indicates the margin of the muscle of interest and where the fascial layer separates the muscle from surrounding tissue.
